# Delayed antibody dependent enhancement of low passage dengue virus 4 isolates

**DOI:** 10.1186/s13104-015-1381-8

**Published:** 2015-09-02

**Authors:** Nitwara Wikan, Sirikwan Libsittikul, Sutee Yoksan, Prasert Auewarakul, Duncan R. Smith

**Affiliations:** Institute of Molecular Biosciences, Mahidol University, Salaya Campus, 25/25 Phuttamonthol Sai 4, Salaya, Nakorn Pathom 73170 Thailand; Center for Emerging and Neglected Infectious Diseases, Mahidol University, Salaya Campus, 25/25 Phuttamonthol Sai 4, Salaya, Nakorn Pathom 73170 Thailand; Department of Microbiology, Faculty of Medicine, Siriraj Hospital, Mahidol University, 2 Wanglang Road, Bangkoknoi, Bangkok, 10700 Thailand

**Keywords:** Antibody dependent enhancement, Dengue, Infection, U937 cells

## Abstract

**Background:**

The concept of antibody dependent enhancement (ADE) of dengue virus (DENV) infection is a cornerstone of our current understanding of dengue pathogenesis, although some questions as to the mechanism remain, particularly in regards to the behavior of low and high passage virus isolates. This study utilized two low passage DENV 4 isolates and a laboratory adapted DENV 4 isolate to investigate the potential of low passage isolates to undergo ADE.

**Results:**

Little or no ADE of infection was observed on day 2 post infection with low passage isolates, while high enhancement of infection was seen with the laboratory adapted virus. However, both of the low passage isolates showed high levels of infection (60–100 %) by day 5 post infection.

**Conclusions:**

These results show that low passage DENV 4 viruses undergo ADE mediated infection, but that the process is significantly temporally delayed as compared to laboratory adapted DENV 4.

**Electronic supplementary material:**

The online version of this article (doi:10.1186/s13104-015-1381-8) contains supplementary material, which is available to authorized users.

## Background

Dengue virus is the most important mosquito transmitted human viral disease worldwide. Each year there are believed to be some 400 million cases of infection, of which some 100 million show some symptoms [1]. Symptomatic infections result in a broad spectrum of disease, ranging from a mild undifferentiated fever, to dengue fever to more severe presentations of dengue hemorrhagic disease and dengue shock syndrome [2]. Fatalities can result from infection with dengue virus, although these are relatively infrequent where adequate hospitalization facilities and appropriate expertise are available [3].

The species *Dengue virus* (Family Flaviviridae, Genus *Flavivirus*) comprises four distinct viruses termed Dengue virus (DENV) 1–4. Primary infection in a naïve individual results in the generation of broad and robust acquired immunity, offering long term, and possibly lifelong immunity to the infecting DENV, however, only transient immunity is generated against infections with heterotypic DENVs [3]. Importantly some antibodies raised against the initial DENV can recognize but not neutralize a heterotypic virus and these DENV:non-neutralizing antibody complexes can be internalized into Fc receptor bearing cells such as monocytes and macrophages [2] cells that are normally either not susceptible or only poorly susceptible to direct infection with DENV. This process, called antibody dependent enhancement (ADE) of infection was largely uncovered by the work of Halstead and colleagues who observed that sub-neutralizing levels of antibodies could trigger this process [2–5]. In particular it is believed that ADE expands the available mass of target cells as well as directly leading to infection of the main immune system regulators resulting in a more severe presentation, and several studies have highlighted the relationship between second infections with a heterotypic virus and disease severity [6–8]. The concept of ADE underpins much of our understanding of the pathogenesis of DENV infections, and plays a major role in shaping vaccine development where efforts focus on generating a uniform and equal immune response to all four DENVs to avoid potential problems of under vaccinated individuals having severe disease as a consequence of an acquired natural infection [9].

Recently, the concept of ADE of infection was partly challenged by Chaichana and colleagues who showed that directly isolated viruses from DENV patients showed little or no ADE of infection in vitro, while laboratory adapted viruses underwent significant ADE of infection using serum or plasma of the same patients [10]. The authors concluded that the source of the virus was an important factor in ADE, and moreover that the ADE of infection seen with laboratory adapted viruses in vitro was a consequence of repeated passage of the virus [10]. We recently noted a similar phenomenon with low passage (as opposed to the unpassaged viruses of Chaichana and colleagues) isolates of DENV 4. Using an optimized protocol with a monoclonal antibody we found that conditions that resulted in high ADE of infection of U937 cells with a laboratory adapted virus resulted in little or no infection with low passage viruses [11]. The publication of the Chaichana paper [10] who did not look at DENV 4 in their study prompted the current study in which we conducted a more complete analysis than our preliminary study [11] to try to find why low passage DENV 4s apparently failed to undergo ADE of infection. Surprisingly, we show that low passage DENV 4 isolates do indeed undergo ADE of infection, but that this is significantly temporally delayed as compared to laboratory adapted viruses.

## Results and discussion

**Fig. 1:**
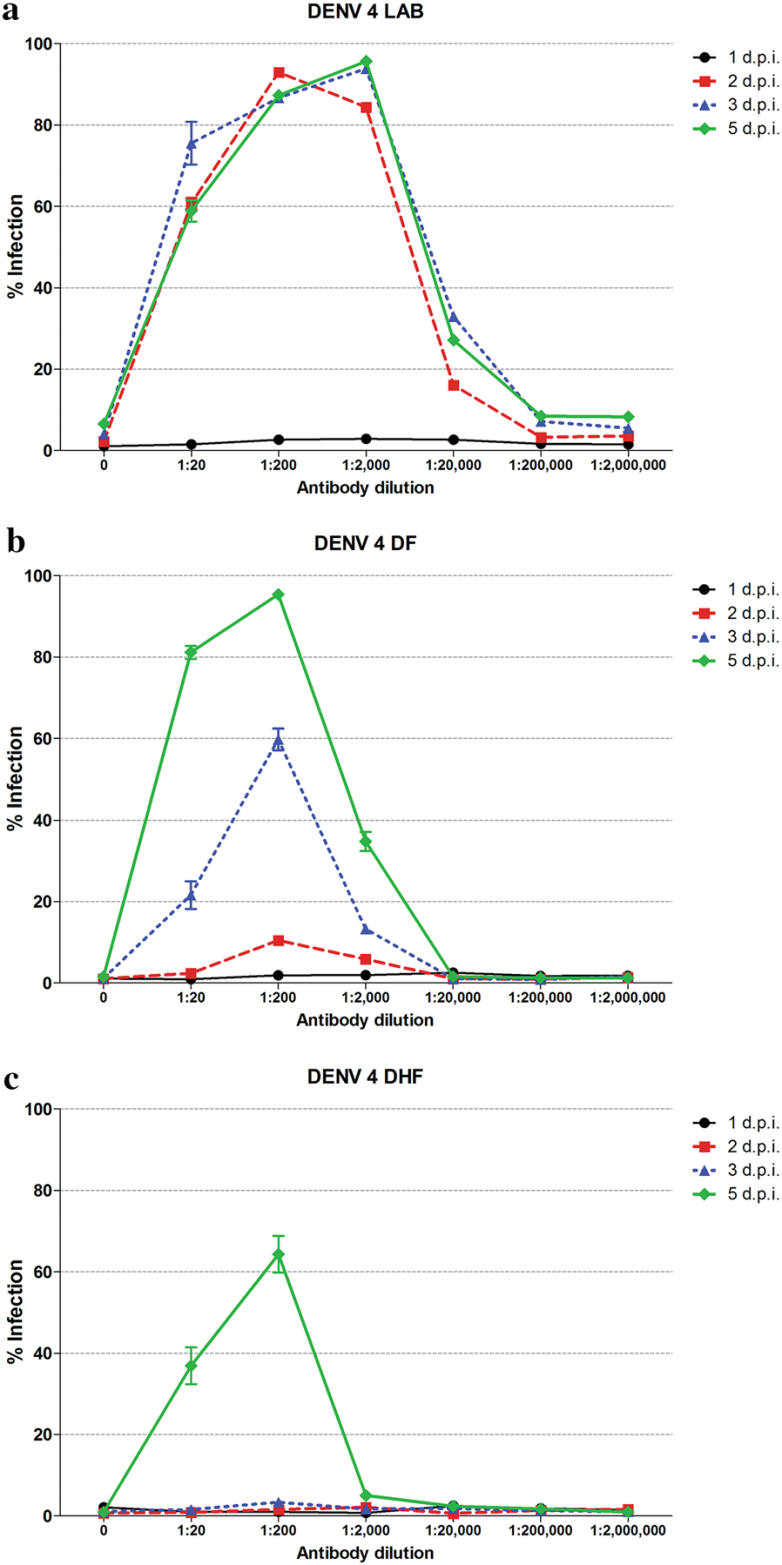
ADE of infection of U937 cells with high and low passage DENVs. Antibody–virus complexes were generated with **a** DENV 4 LAB, **b** DENV 4 DF and **c** DENV 4 DHF and different dilutions of HB 114 antibodies before incubation with U937 cells and subsequent culture for 1–5 days post infection. The degree of infection was determined by flow cytometry. Experiment was undertaken independently in triplicate and *error bars* show SEM

Undifferentiated U937 cells are highly susceptible to ADE mediated DENV infection, but are poorly susceptible to direct DENV infection [12, 13]. Previous studies have suggested that low passage DENVs or DENVs isolated directly from patients do not undergo ADE, or undergo ADE only poorly in comparison to laboratory adapted viruses [10, 11]. To investigate this further, three DENV 4 isolates were selected, one of which was a high passage laboratory (LAB) adapted virus (DENV 4 LAB), one a DENV 4 isolated from a dengue fever (DF) patient and passaged three times in C6/36 (DENV 4 DF) and one a DENV 4 isolated from a dengue hemorrhagic fever (DHF) patient and passaged three times in C6/36 (DENV 4 DHF). Under standard conditions, these three viruses were incubated with either no antibody or increasing dilutions of an antibody produced by hybridoma HB 114, which we have previously shown to be able to mediate ADE with DENV 4 LAB [12]. Cells were incubated under standard conditions and collected on days 1, 2, 3 and 5 post infection before analysis by flow cytometry to determine the degree of infection. Results (Fig. 1) show high levels infection enhancement with DENV 4 LAB from day 2 post infection, with maximum enhancement observed with antibody dilutions between 1:200 and 1:2000, consistent with our previous observations [12]. Significantly, both DENV 4 DF and DENV 4 DHF showed little or no ADE on day 2 post infection, but by day 3 (DENV 4 DF) or day 5 (DENV 4 DHF) post infection, cells infected with low passage isolates showed high degrees of infection, with maximum enhancement being observed with antibody dilutions of 1:200. Notably, cells infected with DENV 4 DF showed nearly 100 % infection by day 5 post infection, while cells infected with DENV 4 DHF showed approximately 60 % infection.

These results suggest that either the low passage isolates are entering the cells during the infection period but are undergoing significantly delayed replication, or that the infection process itself is delayed as compared to laboratory adapted viruses. The concept of delayed replication has been previously established with West Nile virus [14], and it is believed that virulent viruses replicate more slowly to evade innate immune detection, while attenuated viruses undergo rapid replication triggering a stronger innate immune response.

**Fig. 2:**
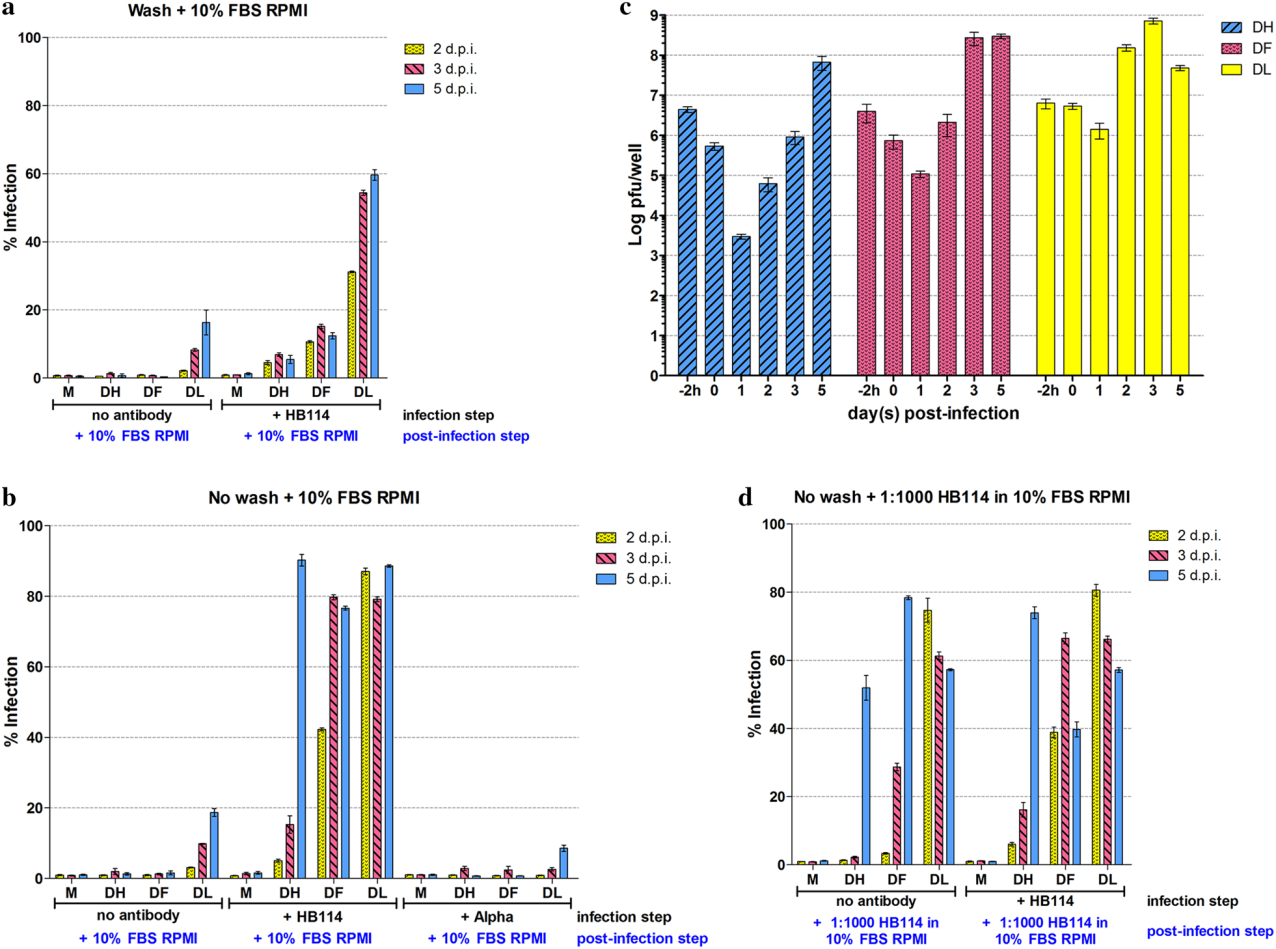
Analysis of ADE infection of U937 cells with high and low passage DENVs. DENV 4 LAB, DENV 4 DF or DENV 4 DHF were incubated with a 1:200 dilution of monoclonal antibody HB114 (+HB 114) or with an isotype matched anti-alphavirus antibody (+alpha) control as appropriate, or with an equivalent volume of medium before incubation with U937 cells for 2 h after which cells were **a** washed with PBS and incubated with normal growth media (RPMI supplemented with 10 % FBS) with daily addition of normal medium to the cells, or **b** incubated with normal growth media with daily addition of normal medium to the cells or **d** incubated with normal growth media with daily addition of normal growth medium containing a 1:1000 dilution of HB 114 antibodies. Percentage infection was determined by flow cytometry and all experiments were undertaken independently in triplicate. *Error bars* show SEM *M* mock infection, DH (DENV 4 DHF) DF (DENV 4 DF), DL (DENV 4 LAB). **c** Plaque assay of supernatants from (**b**) assayed on days “0” (immediately after infection) 1, 2, 3 and 5 post infection. Input virus titer (−2 h) is also displayed. Plaque assays were undertaken independently in triplicate with duplicate assay of titer

To address this issue, an optimized condition of ADE of multiplicity of infection (m.o.i.) 1 and a 1:200 dilution of antibody was selected and after the 2 h incubation of cells with the virus–antibody complex, cells were washed to remove un-internalized virus complexes and subsequently incubated in standard medium. A marked reduction in the level of DENV 4 LAB infection was seen, but only minimal levels (<20 %) of infection were seen with the two low passage isolates (Fig. 2a), even by day 5 post infection. When the washing step was omitted, high levels of infection were again seen with DENV 4 DF by day 3 and DENV 4 DHF by day 5 (Fig. 2b). Control experiments using an isotyped matched anti-alphavirus antibody showed no evidence of infection above levels seen with no antibody (Fig. 2b). These experiments show that it is not virus replication that is delayed in the low passage isolates, but the infection process itself. In parallel with these experiments, cells were incubated with virus but with no antibody. These are effectively an assessment of the ability of the viruses to directly enter into U937 cells in the absence of antibody–virus complex formation. Some (<20 %) direct infection was observed with DENV 4 LAB was observed, but no direct entry was seen with the low passage isolates.

To provide further confirmation of the delayed infection of U937 cells with low passage isolates, the supernatants from the infections with the washing step omitted, but antibody included (as shown in Fig. 2b) were assayed for infectious virus titer on days 0 to day 5 post infection by standard plaque assay. Results (Fig. 2c) showed that the virus titer for DENV 4 LAB was over input titer (as assayed on “day 0”) by day 2 post infection, virus titers for DENV 4 DF and DENV 4 DHF were over input virus on days 3 and 5 respectively (Fig. 2c). These results are consistent with the data for percentage of infection as determined by flow cytometry (Fig. 2b).

In a final series of experiments, the infection was again repeated without washing after incubation of the virus or virus–antibody complex with cells, and this time a 1:1000 dilution of antibody was added daily to all infections. This included cells that were infected with virus that did not have the initial antibody-complex formation step (i.e. direct infection), but antibody was added daily to the culture medium. Results (Fig. 2d) showed high levels of infection in all samples. Levels of infection of DENV 4 LAB and DENV 4 DF apparently dropped after reaching a peak, but this was due to increasing numbers of dead cells in the culture media (although this was not formally evaluated). Importantly, high levels of infection were also seen in the experiments in which no antibody had been added during the original infection period, but only supplemented post infection (Fig. 2d).

Combined, these results clearly show that low passage DENV 4 isolates undergo ADE of infection in the presence of a suitable antibody, but that the process is somewhat temporally delayed with low passage isolates as compared to laboratory adapted viruses. Adding antibody daily after the initial infection markedly improved the level of infection, and suggests that the standard infection protocol of allowing antibody–virus complex to form prior to infection is not necessary. Although our study uses low passage DENV 4 isolates and not unpasssaged isolates as used by Chaichana and colleagues [10], our results with DENV 4 DHF, in which no infection was seen until day 5 would be consistent with their observations which were taken to only day 3 post infection. The somewhat earlier infection seen with DENV 4 DF would support their observation that the source of the virus is important. Previous studies have suggested that ADE of infection has both an extrinsic and an intrinsic component [15]. Extrinsic ADE is the increased uptake of virus/antibody complexes, while intrinsic ADE is the suppression of innate immune responses [16]. In this case, it is possible that the low passage DENV 4 isolates do not suppress innate immunity as well as the high passage isolate, leading to the comparatively delayed infection profile.

## Conclusions

While limited in scope, this study has shown that there is significant variation in the time profile of ADE infection of different DENVs. The mechanism requires further investigation, but the study highlights the need to look at longer time points when using low passage viruses.

## Methods

### Ethics

The two low passage DENV isolates described in this study were originally collected in 2006 after written individual informed consent in a study approved by the Ethical Review Committee for Research in Human Subjects, Ministry of Public Health, Thailand [17, 18].

### Cells and viruses

Vero (ATCC Cat No. CCL-81), LLC-MK2 (ATCC Cat No. CCL-7), U937 (ATCC Cat No. CRL-1593.2) and C6/36 [19] cells were cultured exactly as described previously [20]. Laboratory adapted DENV 4 (strain 1036; GenBank accession KM519590) was propagated exactly as described previously [12]. The virus used had been passaged 5 times in mammalian cells and 4 times in C6/36 cells before use in this study. Two strains of DENV 4 used were isolated from a dengue fever patient and a dengue hemorrhagic fever patient DENV 4DF (GenBank accession: KM519591) and DENV 4DHF (GenBank accession: KM519592) respectively. These viruses were passaged three times in C6/36 cells before use in this study. Both patients were serologically identified as having secondary infections. These viruses were passaged three times in C6/36 before being stored at −80 °C until required. Virus titer of all viruses was determined by standard plaque assay on LLC-MK2 cells, essentially as described elsewhere [21].

### ADE infection protocol and evaluation of infection

Stock DENV at a m.o.i. of 1 was incubated with either RPMI or with increasing dilutions of a monoclonal antibody purified in house from hybridoma HB114 [22] for 1 h with constant agitation at 4 °C to generate virus antibody complexes which were then added to 3 × 10^6^ U937 cells in RPMI medium followed by incubation at 37 °C for a further 2 h with constant agitation. Cells were then resuspended to a dilution of 3 × 10^5^ cells mL^−1^ in RPMI buffer supplemented with 10 % fetal bovine serum (RPMI-10 %) before incubation at 37 °C. In some cases cells were washed with PBS before re-suspension. Fresh RPMI-10 % was added daily which in some cases was additionally supplemented with monoclonal antibodies produced by hybridoma HB114 [22] at a 1:1000 dilution. Control experiments using an isotype matched anti-alphavirus antibody (sc-58088; Santa Cruz Biotechnology, Inc. Santa Cruz, CA) were additionally undertaken.

Flow cytometry to determine percentage infection was undertaken essentially as described elsewhere [12]. Briefly, approximately 1 × 10^6^ cells were collected by centrifugation at 1000×*g* for 5 min, blocked with 10 % normal goat serum (Gibco BRL, Gaithersburg, MD) in phosphate buffer saline (PBS) and left on ice for 30 min, fixed in 4 % paraformaldehyde (Sigma-Aldrich Co., St. Louis, MO) in PBS for 20 min and permeabilized with 0.2 % Triton X-100 (Calbiochem, Merck KGaA, Darmstadt, Germany) in PBS for 10 min. After washing once with 1 % BSA in PBS, cells were stained for intracellular expression of dengue antigen using a 1:20 dilution of monoclonal antibodies from hybridoma HB114 [22] overnight at 4 °C. Following washing twice with 1 % BSA in PBS a 1:40 dilution of a polyclonal goat anti-mouse IgG conjugated to FITC (KPL, Gaithersburg, MD) was used as a secondary antibody and cells were incubated for 1 h and then washed twice as previously. The washed cells were resuspended in 100 µl of PBS and analyzed by flow cytometry (FACS Calibur, BD Biosciences, San Jose, CA) using CELLQuest™ software (BD). All experiments were undertaken independently in triplicate. Infected cells were gated as M1 (Raw data is provided in Additional file 1).

## Additional file

Additional file 1: Raw M1 gate values for all experiments.

## Abbreviations

ADE: antibody dependent enhancement
DENV: dengue virus
DF: dengue fever
DHF: dengue hemorrhagic fever

## References

[1] Bhatt S, Gething PW, Brady OJ, Messina JP, Farlow AW, Moyes CL, Drake JM, Brownstein JS, Hoen AG, Sankoh O (2013). The global distribution and burden of dengue. Nature.

[2] Halstead SB (1989). Antibody, macrophages, dengue virus infection, shock, and hemorrhage: a pathogenetic cascade. Rev Infect Dis.

[3] Gubler DJ (1998). Dengue and dengue hemorrhagic fever. Clin Microbiol Rev.

[4] Halstead SB, O’Rourke EJ (1977). Dengue viruses and mononuclear phagocytes. I. Infection enhancement by non-neutralizing antibody. J Exp Med.

[5] Halstead SB, Porterfield JS, O’Rourke EJ (1980). Enhancement of dengue virus infection in monocytes by flavivirus antisera. Am J Trop Med Hyg.

[6] Guzman MG, Kouri GP, Bravo J, Soler M, Vazquez S, Morier L (1990). Dengue hemorrhagic fever in Cuba, 1981: a retrospective seroepidemiologic study. Am J Trop Med Hyg.

[7] Rothman AL (2010). Cellular immunology of sequential dengue virus infection and its role in disease pathogenesis. Curr Top Microbiol Immunol.

[8] Sangkawibha N, Rojanasuphot S, Ahandrik S, Viriyapongse S, Jatanasen S, Salitul V, Phanthumachinda B, Halstead SB (1984). Risk factors in dengue shock syndrome: a prospective epidemiologic study in Rayong, Thailand. I. The, 1980 outbreak. Am J Epidemiol.

[9] Endy TP (2014). Human immune responses to dengue virus infection: lessons learned from prospective cohort studies. Front Immunol.

[10] Chaichana P, Okabayashi T, Puiprom O, Sasayama M, Sasaki T, Yamashita A, Ramasoota P, Kurosu T, Ikuta K (2014). Low levels of antibody-dependent enhancement in vitro using viruses and plasma from dengue patients. PLoS One.

[11] Rungruengphol C, Roytrakul S, Smith DR. Susceptibility of monocytic cells to dengue virus infection. In: 27th national graduate research conference; 28th February–1 March; 27th national graduate research conference, Naresuan University, Phitsanulok, Thailand; 2013.

[12] Klomporn P, Panyasrivanit M, Wikan N, Smith DR (2011). Dengue infection of monocytic cells activates ER stress pathways, but apoptosis is induced through both extrinsic and intrinsic pathways. Virology.

[13] O’Sullivan MA, Killen HM (1994). The differentiation state of monocytic cells affects their susceptibility to infection and the effects of infection by dengue virus. J Gen Virol.

[14] Scherbik SV, Pulit-Penaloza JA, Basu M, Courtney SC, Brinton MA (2013). Increased early RNA replication by chimeric West Nile virus W956IC leads to IPS-1-mediated activation of NF-kappaB and insufficient virus-mediated counteraction of the resulting canonical type I interferon signaling. J Virol.

[15] Halstead SB, Mahalingam S, Marovich MA, Ubol S, Mosser DM (2010). Intrinsic antibody-dependent enhancement of microbial infection in macrophages: disease regulation by immune complexes. Lancet Infect Dis.

[16] Ubol S, Phuklia W, Kalayanarooj S, Modhiran N (2010). Mechanisms of immune evasion induced by a complex of dengue virus and preexisting enhancing antibodies. J Infect Dis.

[17] Sabchareon A, Sirivichayakul C, Limkittikul K, Chanthavanich P, Suvannadabba S, Jiwariyavej V, Dulyachai W, Pengsaa K, Margolis HS, Letson GW (2012). Dengue infection in children in Ratchaburi, Thailand: a cohort study. I. Epidemiology of symptomatic acute dengue infection in children, 2006–2009. PLoS Negl Trop Dis.

[18] Yoksan S, Tubthong K, Kanitwithayanun W, Jirakanjanakit N (2009). Laboratory assays and field dengue vaccine evaluation at Ratchaburi province, Thailand: a preliminary result. J Clin Virol.

[19] Singh KRP (1967). Cell cultures derived from larvae of *Aedes albopictus* (Skuse) and *Aedes aegypti* (L.). Curr Sci.

[20] Wikan N, Sakoonwatanyoo P, Ubol S, Yoksan S, Smith DR (2012). Chikungunya virus infection of cell lines: analysis of the East, central and South African lineage. PLoS One.

[21] Sithisarn P, Suksanpaisan L, Thepparit C, Smith DR (2003). Behavior of the dengue virus in solution. J Med Virol.

[22] Henchal EA, Gentry MK, McCown JM, Brandt WE (1982). Dengue virus-specific and flavivirus group determinants identified with monoclonal antibodies by indirect immunofluorescence. Am J Trop Med Hyg.

